# Topoisomerase I Inhibitors, Shikonin and Topotecan, Inhibit Growth and Induce Apoptosis of Glioma Cells and Glioma Stem Cells

**DOI:** 10.1371/journal.pone.0081815

**Published:** 2013-11-26

**Authors:** Feng-Lei Zhang, Ping Wang, Yun-Hui Liu, Li-bo Liu, Xiao-Bai Liu, Zhen Li, Yi-Xue Xue

**Affiliations:** 1 Department of Neurobiology, College of Basic Medicine, China Medical University, Shenyang, Liaoning Province, People’s Republic of China; 2 Institute of Pathology and Pathophysiology, China Medical University, Shenyang, Liaoning Province, People’s Republic of China; 3 Department of Neurosurgery, Shengjing Hospital of China Medical University, Shenyang, Liaoning Province, People’s Republic of China; 4 The 96^th^ Class, 7-Year Program, China Medical University, Shenyang, Liaoning Province, People’s Republic of China; Columbia University, United States of America

## Abstract

Gliomas, the most malignant form of brain tumors, contain a small subpopulation of glioma stem cells (GSCs) that are implicated in therapeutic resistance and tumor recurrence. Topoisomerase I inhibitors, shikonin and topotecan, play a crucial role in anti-cancer therapies. After isolated and identified the GSCs from glioma cells successfully, U251, U87, GSCs-U251 and GSCs-U87 cells were administrated with various concentrations of shikonin or topotecan at different time points to seek for the optimal administration concentration and time point. The cell viability, cell cycle and apoptosis were detected using cell counting kit-8 and flow cytometer to observe the inhibitory effects on glioma cells and GSCs. We demonstrated that shikonin and topotecan obviously inhibited proliferation of not only human glioma cells but also GSCs in a dose- and time-dependent manner. According to the IC50 values at 24 h, 2 μmol/L of shikonin and 3 μmol/L of topotecan were selected as the optimal administration concentration. In addition, shikonin and topotecan induced cell cycle arrest in G0/G1 and S phases and promoted apoptosis. The down-regulation of Bcl-2 expression with the activation of caspase 9/3-dependent pathway was involved in the apoptosis process. Therefore, the above results showed that topoisomerase I inhibitors, shikonin and topotecan, inhibited growth and induced apoptosis of GSCs as well as glioma cells, which suggested that they might be the potential anticancer agents targeting gliomas to provide a novel therapeutic strategy.

## Introduction

Glioma is one of the most common malignant brain tumors in adults. Over recent years, more and more studies have pointed out that the fatal nature of glioma is caused by glioma stem cells (GSCs), which are present in glioma. GSCs share many properties of normal stem cells, such as the ability to self-renew, resistance to toxic compounds, asymmetric cell division, and have been postulated to be more resistant to the hypoxic tumor microenvironment [1,2]. GSCs are a potential therapeutic target to solve tumor formation, development and recurrences. 

DNA topoisomerases regulate the topological status of the DNA double helix and induce either single (Topo I)- or double (Topo II)-strand DNA breaks and are thus key enzymes for DNA replication, transcription, recombination and chromatin remodeling [3]. Topo I is a 100 kDa monomeric protein encoded by a single copy gene located on 20q12-13.2 and requires phosphorylation for full expression of its activity [4]. Some reports showed that recurrent human colorectal cancer biopsies and breast cancer stem cells contained significantly higher levels of Topo I than normal tissues [5,6]. Relapsed ovarian cancer and small cell lung cancer have been shown to be sensitive to the topo I inhibitor as well [7,8]. Topo I has become not only an important indicator to evaluate the proliferation state of various malignant cells, but also a privileged target of many chemotherapeutics. Topo I inhibitors can be divided into the Topo I poison and the suppressor types, both of which act specifically at the level of the topoisomerase I-DNA complex and stimulate DNA cleavage. The Topo I poisons, like topotecan, act after the cleavage of DNA by the enzyme and inhibit the religation. The sensitivity of tumor cells to Topo I poisons increases associated with the overexpression of Topo I. In contrast, Topo I suppressors, like shikonin, inhibit binding of topoisomerase I to the DNA cleavage site, thus preventing all subsequent steps in the catalytic cycle. The activity of Topo I suppressors is higher in tumor cells with low-expressed Topo I [9,10]. Thus, these two classes of inhibitor show separate mechanisms in anti-cancer treatment.

Topotecan is a water-soluble camptothecin analog that has shown cytotoxicity toward a variety of tumor types [11]. It can pass through the blood-brain barrier and exhibit the significant activity in treating brain tumors [12,13]. Shikonin, an anthraquinone derivative extracted from the root of lithospermum, shows the antitumor effects by inhibiting tumor cell growth and inducing apoptosis [14,15]. Apoptosis manifests in two major execution programs downstream of the death signal: the caspase pathway and organelle dysfunction, of which mitochondrial dysfunction is best characterized. The B-cell lymphoma/leukemia-2 (Bcl-2) family members reside upstream of irreversible cellular damage and focus much of their efforts at the level of mitochondria. They are often misappropriated in many cancers, including lung carcinoma, lymphoma, and GBM, and consequently emerged as therapeutic targets [16,17]. Caspases could be activated through Apaf-1/cytochrome *c* in the mitochondrial-initiated pathway or directly by activation of cell surface death receptors. Activated caspase-9 will then cleave and activate downstream caspases such as caspase-3, -6, and -7. Shikonin had been found to induce human bladder cancer cells apoptosis by promoting the activation of caspase-3 [18]. However, the effects of Topo I inhibitors, shikonin and topotecan, on GSCs have not been clarified yet. The main purpose of this study is to investigate the possible effects of shikonin and topotecan on GSCs as well as the glioma cells and the potential mechanisms.

## Materials and Methods

### Cell Culture

The human U251 and U87 glioma cells were purchased from Nanjing KGI Biotechnology Company and were grown in DMEM/F12 medium (1:1, Hyclone) supplemented with 10% fetal bovine serum (invitrogen) in a humidified atmosphere of 5% CO_2_ at 37°C. To obtain the GSCs-U251 and GSCs-U87, the U251 and U87 glioma cells in logarithmic growth phase were dissociated enzymatically and switched into a serum free medium DMEM/F12 containing 20 ng/ml of EGF, 20 ng/ml of FGF-2 (PeproTech) and 2% B27 Neuro Mix (invitrogen). These cells were incubated to form the primary sphere, added with a 20% volume of fresh medium twice a week. When the diameter reached to 200–300 μm, the suspended cellular spheroids were harvested, dissociated into single cell with trypsin-EDTA and re-seeded. Simultaneously, the monolayer cells left in the flasks were transferred into medium without growth factor but permissive for differentiation.

### Glioma Stem Cells Identification

Cellular spheroids and differentiated cells were grown in precoated chamber slides. After washing with PBS, the GSCs were fixed in 4% paraformaldehyde (Sigma-Aldrich), permeabilized using 0.1% Triton-X100 (Sigma-Aldrich) for 20 min and blocked in 5% BSA (Sigma-Aldrich) for 1 h at room temperature. Then the GSCs were immunostained with CD133 (1:200, Santa Cruz), nestin (1:300, Santa Cruz), β-tubulin III (1:500, Abcam) and glial fibrillary acidic protein (GFAP, 1:500, Abcam) for 45 min in darkness. Subsequent visualization was performed with TRITC-conjugated secondary antibody (1:1000, Santa Cruz) and FITC-conjugated secondary antibody (1:1000, Santa Cruz) for 30 min at room temperature and the nuclei were stained with DAPI (1:500, Santa Cruz). Fluorescence images were captured with fluorescence microscope (Olympus BX51).

### Flow Cytometry Analysis of CD133 Positive Cells

For flow cytometry analysis, GSCs enriched in DMEM/F12 medium plus growth factors were dissociated, washed, and incubated with PE-conjugated CD133 antibody (Milteny Biotech) at a dilution of 1:10 in phosphate-buffered saline (PBS)–bovine serum albumin (BSA) for 30 min at 4°C. For the control, cells were incubated with an isotype IgG antibody. Expression level analysis was done on FACScan and FACSAria, respectively (BD Bioscience).

### Growth Inhibition Assays

The U251, U87, GSCs-U251 and GSCs-U87 cells were seeded at a density of 2×10^4^ cells per well in 96-well plates separately, and incubated for 24 h. Cells were administered with shikonin (National Institute for the Control of Pharmaceutical and Biological Products) and topotecan (Sigma-Aldrich). After the treatment, 10 μl of cell counting kit-8 (CCK-8; Dojindo Laboratories, Japan) was added into each well for additional 1-hour incubation at 37°C. The optical density (OD) was read with a microplate reader (Molecular Devices, USA) at 450 nm.

### Cell Cycle Analysis

After treated with shikonin or topotecan for 24 h, the cells (5×10^6^) were harvested and then fixed with 500 μl of 70% cold ethanol for 2 h. The cells were added with 100 μl of RNase and incubated at 37°C for 30 min. Then, 400 μl of PI was added, and the cells were incubated at 4°Cfor 30 min away from light. The samples were immediately subjected to flow cytometer (FACScan, Becton Dickinson). The results were analyzed using CELLQuest 3.0 software (BD, USA). 

### Annexin V-FITC and PI Assay for Flow Cytometry

After treated with shikonin and topotecan for 24 h, the cells (5×10^6^) were collected and resuspended in 200 μl of binding buffer. Annexin V-FITC (10 μl, Beijing Baosai Biotech, China) was added to the cell suspension, followed by incubation at room temperature in the dark for 15 min. After this step, 300 μl of binding buffer and 5 μl of PI were added, and the samples were immediately measured using a flow cytometer. The results were analyzed using CELLQuest 3.0 software. The dots in the lower left quadrant (LL), upper left quadrant (UL), upper right quadrant (UR), and lower right quadrant (LR) represents viable cells (Annexin V−, PI−), damaged cells (Annexin V−, PI+), cells in late apoptosis (Annexin V+, PI+) and cells in early apoptosis (Annexin V+, PI−), respectively.

### Western Blot

Cells treated with shikonin, topotecan or cisplatin (Sigma-Aldrich, USA) for 24 h were collected and lysed in RIPA buffer. Equal amounts of proteins (30 μg) were separated by SDS-PAGE gels and were transferred to a PVDF membrane, blocked with 5% nonfat dry milk in TBST. The blots were incubated with the primary antibody of p21 (1:500, Santa Cruz), Bcl-2 (1:500, Santa Cruz), cleaved caspase-9 (Asp330, 1:1000, Cell Signaling), cleaved caspase-3 (Asp175, 1:1000, Cell Signaling) and an HRP-conjugated secondary antibody solution. After subsequent washes, immunoblots were visualized by the ECL plus western blotting detection system (Santa Cruz Biotechnology). Autoradio-graphic images were scanned using Chemi Imager 5500 V2.03 software, and the integrated density values were calculated by computerized image analysis system (Fluor Chen 2.0) and normalized with that of β-actin.

### Statistical Analysis

Dates were presented as the mean±SD for each group. Data were analyzed using the SPSS 13.0 software. One-way ANOVA followed by Dunnett-t test was used to determine significant differences between groups. A statistical significance was inferred at *P*<0.05.

## Results

### Identification of GSCs from U251 and U87 cells

**Figure 1:**
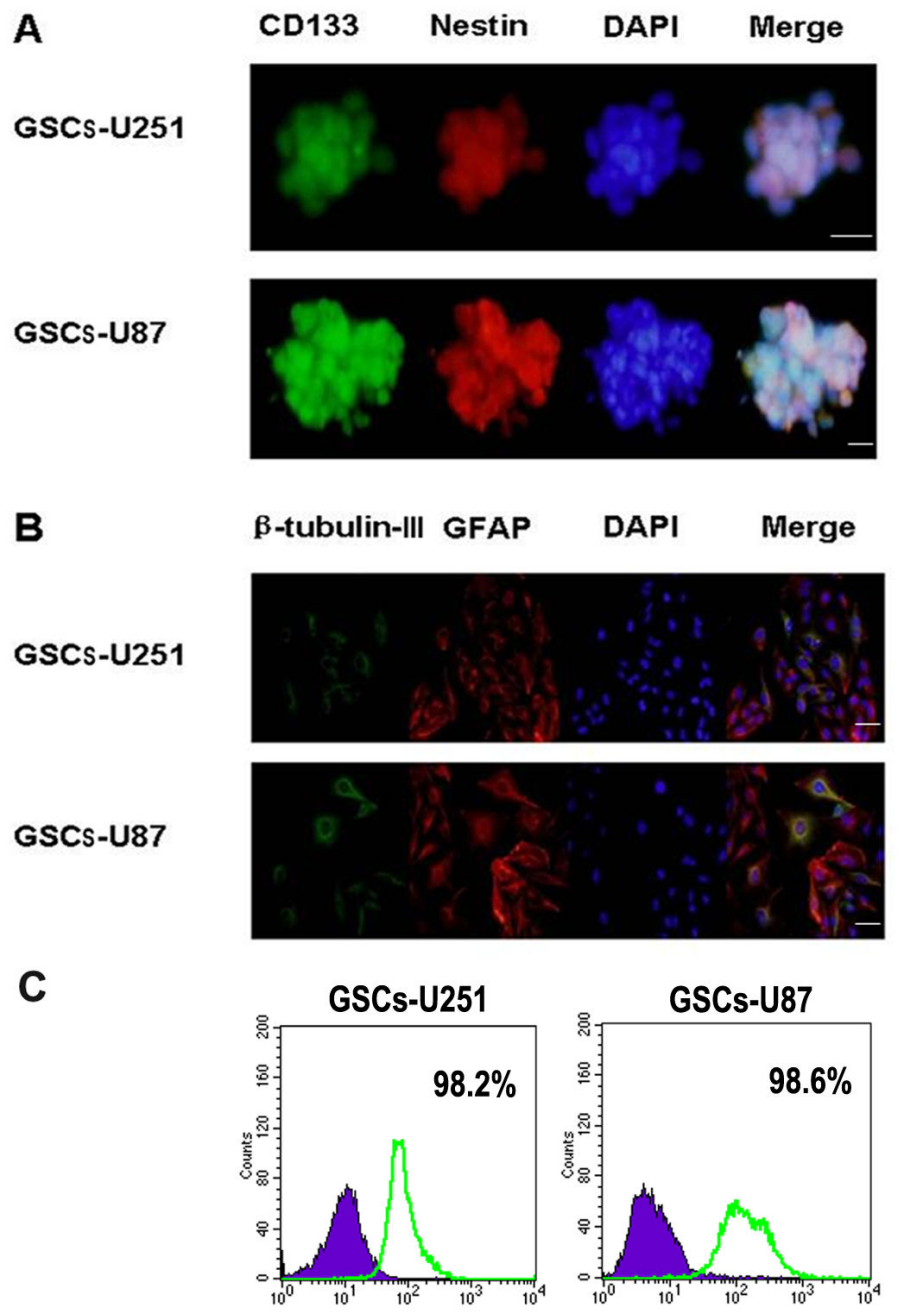
Identification of GSCs-U251 and GSCs-U87. (A) Immunocytochemical staining of the neural progenitor markers nestin (red) and the cancer stem cell marker CD133 (green) in GSCs-U251 and GSCs-U87 cells. Scale bar: 20 μm. (B) Immunocytochemical staining was performed to assess multilineage differentiation of β-tubulin-III positive neurons (green) and GFAP-positive astrocytes (red). Scale bar: 25 μm. (C) The percentage of CD133^+^ cells in the isolated GSCs-U251 and GSCs-U87 analyzed by flow cytometry.

U251 and U87 cells growing exponentially formed sphere-shaped colonies gradually even when seeded at a low density, which indicated a strong self-renewal capacity. The cells within the sphere were positive to neural stem cell markers CD133 (Figure 1A; green) and nestin (Figure 1A; red), which were involved in self-renewal and proliferation of stem cells. The assay of multi-lineage differentiation capacity of cells within the sphere was demonstrated by culturing the cells in differentiation-inducing culture medium. These cells showed typical morphological differentiation towards neuronal and astrocytic lineages, identified as β-tubulin-III positive neurons (Figure 1B; green) and GFAP-positive astrocytes (Figure 1B; red), respectively. To determine the percentage of cells with the unique characteristics of stem cells, quantitative analysis of CD133^+^ cells were performed by flow cytometry. The result showed that the isolated GSCs using sphere culture contained 98.2% and 98.6% CD133^+^ cells in GSCs-U251 and GSCs-U87, respectively (Figure 1C).

### Shikonin or Topotecan Inhibits GSCs Growth

**Figure 2:**
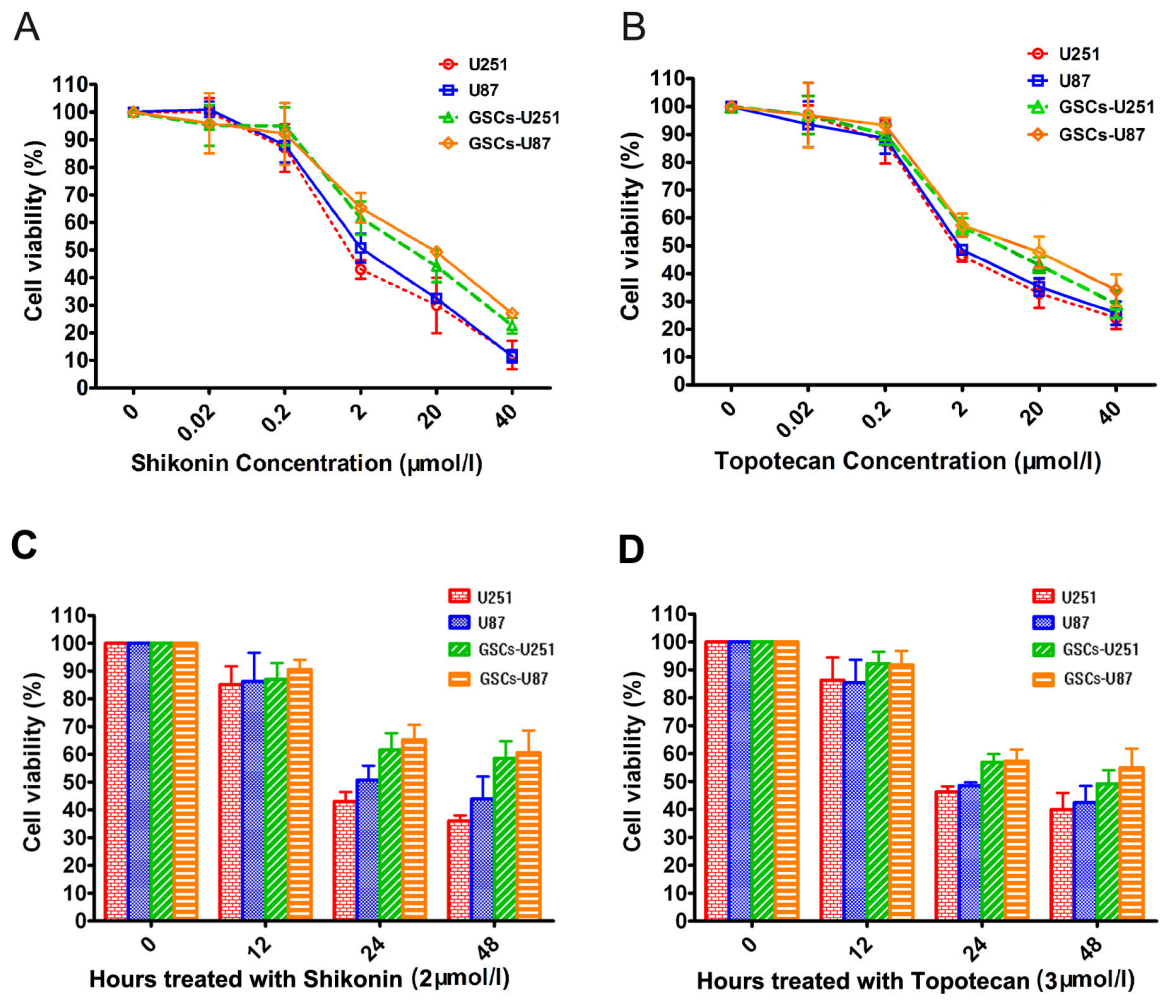
Shikonin or topotecan inhibit U251, U87, GSCs-U251 and GSCs-U87 cells proliferation in a dose- and time-dependent manner. U251, U87, GSCs-U251 and GSCs-U87 cells were treated with different concentrations of shikonin (A) and topotecan (B) for 24 h, and cell viability were assayed by CCK-8. U251, U87, GSCs-U251 and GSCs-U87 cells were treated with 2 μmol/L of shikonin (C) and 3 μmol/L of topotecan (D) for different time points.

After treated with five different concentrations (0.02, 0.2, 2, 20, 40 μmol/L) of shikonin or topotecan for 24 hours, the cell viability of U251, U87, GSCs-U251, GSCs-U87 cells were assessed using the CCK-8 assay. Results showed that the cell viability was inhibited by shikonin or topotecan in a dose-dependent manner (Figure 2 A and B). 2, 20 and 40 μmol/L of shikonin or topotecan obviously inhibited the cell viability compared with the control groups. The IC50 values of shikonin at 24 h were 1.84±0.34 μmol/L of U251 cells, 2.02±0.44 μmol/L of U87 cells, 3.72±0.27 μmol/L of GSCs-U251 and 3.95±0.19 μmol/L of GSCs-U87. Therefore, 2 μmol/L of shikonin was selected as the optimal administration concentration according to the IC50 data in the subsequent experiments. The IC50 values of topotecan at 24 h were 2.73±0.25 μmol/L of U251 cells, 2.95±0.23 μmol/L of U87 cells, 5.46±0.41 μmol/L of GSCs-U251 and 5.95±0.24 μmol/L of GSCs-U87. Thus 3 μmol/L of topotecan was selected as the optimal administration concentration in the subsequent experiments. 

After treated with shikonin (2 μmol/L) or topotecan (3 μmol/L) for 12, 24 and 48 hours, the viability of U251, U87, GSCs-U251 and GSCs-U87 cells was inhibited in a time-dependent manner (Figure 2 C and D). Shikonin or topotecan obviously inhibited the proliferation of the above cells at 24 h, therefore, the 24-hour administration time was selected as the optimal time point in the subsequent experiments.

### Shikonin or Topotecan Induces GSCs Cell Cycle Arrest in G0/G1 and S Phases and Apoptosis

**Figure 3:**
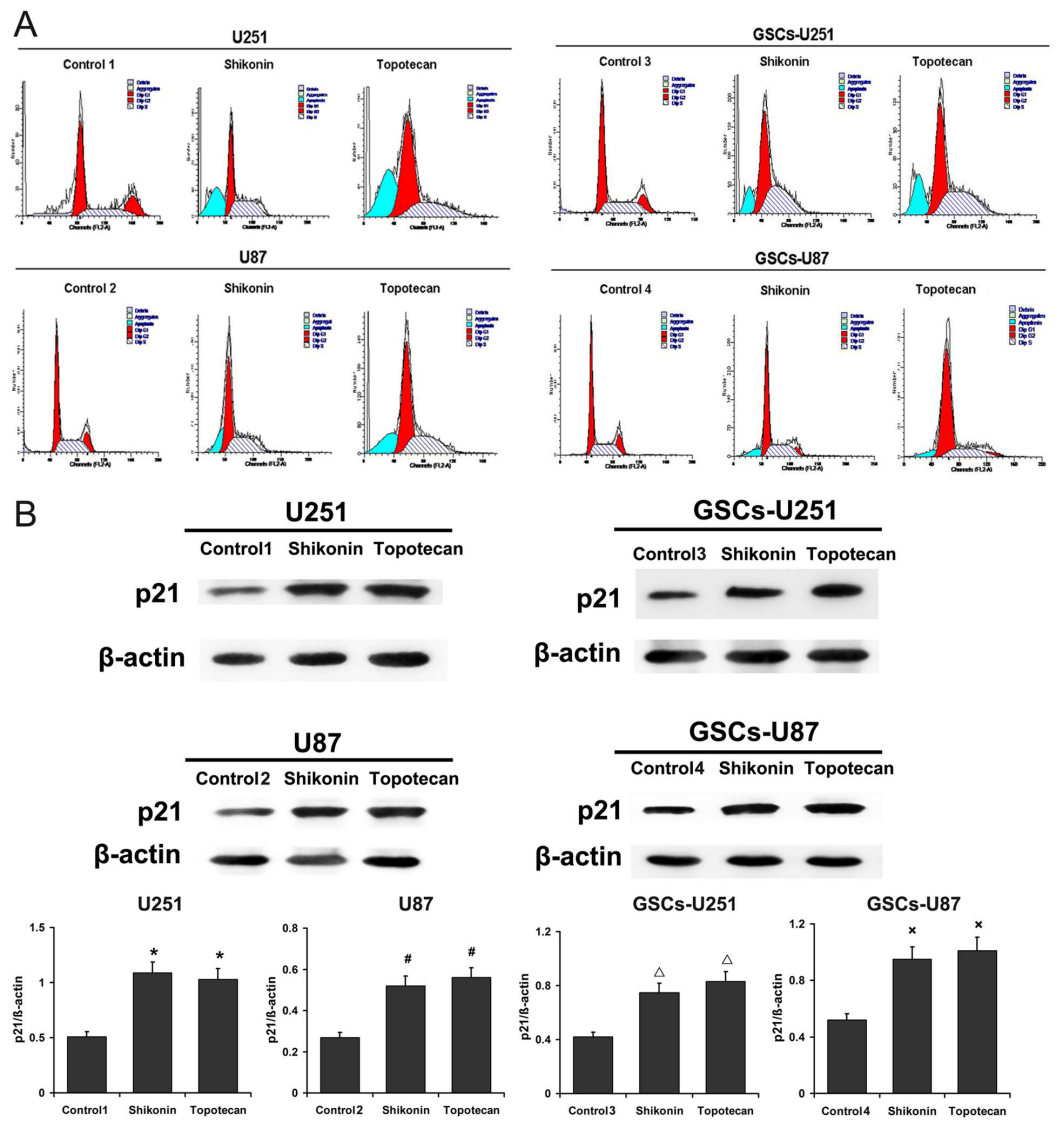
Effects of shikonin or topotecan on cell cycle distribution. (A) The peak chart of cell cycle of U251, U87, GSCs-U251 and GSCs-U87 cells treated with shikonin or topotecan. (B) The p21 expressing levels and quantitative analysis. Data are given as mean±SD and are representative of three separate experiments. *P<0.01 versus untreated U251 cells. ^***#***^ *P*<0.01 versus untreated U87. ^Δ^ *P*<0.01 versus untreated GSCs-U251. ^×^ *P*<0.01 versus untreated GSCs-U87.

PI staining for flow cytometry was used to detect cell cycle arrest in U251, U87, GSCs-U251 and GSCs-U87 cells treated with shikonin or topotecan for 24 h. As shown in Figure 3A and Table 1, Shikonin or topotecan significantly increased the G0/G1 and S phase cell populations of GSCs as well as the glioma cells (*P*<0.01), accompanied with the decreased G2 phase cell populations (*P*<0.01), which suggested that the cell cycles were arrested at G0/G1 and S phase induced by shikonin or topotecan. The expressing levels of cyclin-dependent kinase inhibitor p21 were also analyzed by western blot (Figure 3B). The results indicated that the p21 protein levels were significantly up-regulated compared with control groups after the treatment of shikonin or topotecan (*P*<0.01). The G0/G1 phase arrest might be caused by the induction of p21. A sub-G1 cell population could also be seen to the left of the G0/G1 peak, which suggested the presence of an apoptotic cell population.

**Table 1:** Modulation of cell cycle and apoptosis by Shikonin or Topotecan.

**Group**	**Sub-group**	**Mean±SD (%**)	**Mean±SD (%**)	**Mean±SD (%**)	**Mean±SD (%**)
		**Sub-G0/G1 phase**	**G0/G1 phase**	**S phase**	**G2/M phase**
**U251**	**Control 1**	0.55±0.89	49.71±2.81	26.40±2.45	21.47±3.04
	**Shikonin**	32.72±1.99**	58.3±1.17*	38.39±3.52**	3.07±1.52**
	**Topotecan**	36.8±2.8**	63.22±2.45**	35.63±1.96*	1.19±1.03**
**U87**	**Control 2**	0.88±1.08	50.99±1.63	30.32±2.45	14.72±1.89
	**Shikonin**	22.95±3.62^##^	58.41±1.62^#^	40.94±2.99^##^	2.20±0.68^##^
	**Topotecan**	24.26±1.55^##^	63.07±4.23^##^	37.07±1.62^#^	1.38±1.44^##^
**GSCs-U251**	**Control 3**	0.79±0.98	51.42±2.37	30.95±2.86	16.87±2.81
	**Shikonin**	13.73±2.05^ΔΔ^	59.35±3.15^Δ^	39.15±2.01^Δ^	2.79±1.73^ΔΔ^
	**Topotecan**	19.59±3.44^ΔΔ^	58.15±1.92^Δ^	40.07±3.08^Δ^	2.33±2.03^ΔΔ^
**GSCs-U87**	**Control 4**	1.44±1.35	50.60±2.08	29.82±2.08	17.69±2.99
	**Shikonin**	13.24±2.69^××^	59.89±3.26^×^	34.84±1.74^×^	8.15±2.57^×^
	**Topotecan**	13.17±3.54^××^	57.06±0.94^×^	35.83±1.81^×^	8.67±2.40^×^

The analysis of cell cycle distribution and apoptosis rate in U251, U87, GSCs-U251 and GSCs-U87. Data is given as mean±SD and are representative of three separate experiments. ^**^ *P*<0.01, ^*^ *P*<0.05 versus control 1; ^##^ *P*<0.01, ^#^ *P*<0.05 versus control 2; ^ΔΔ^ *P*<0.01, ^Δ^ *P*<0.05 versus control 3; ^××^ *P*<0.01, ^×^ *P*<0.05, versus control 4.

### Shikonin or Topotecan Induces GSCs Apoptosis

**Figure 4:**
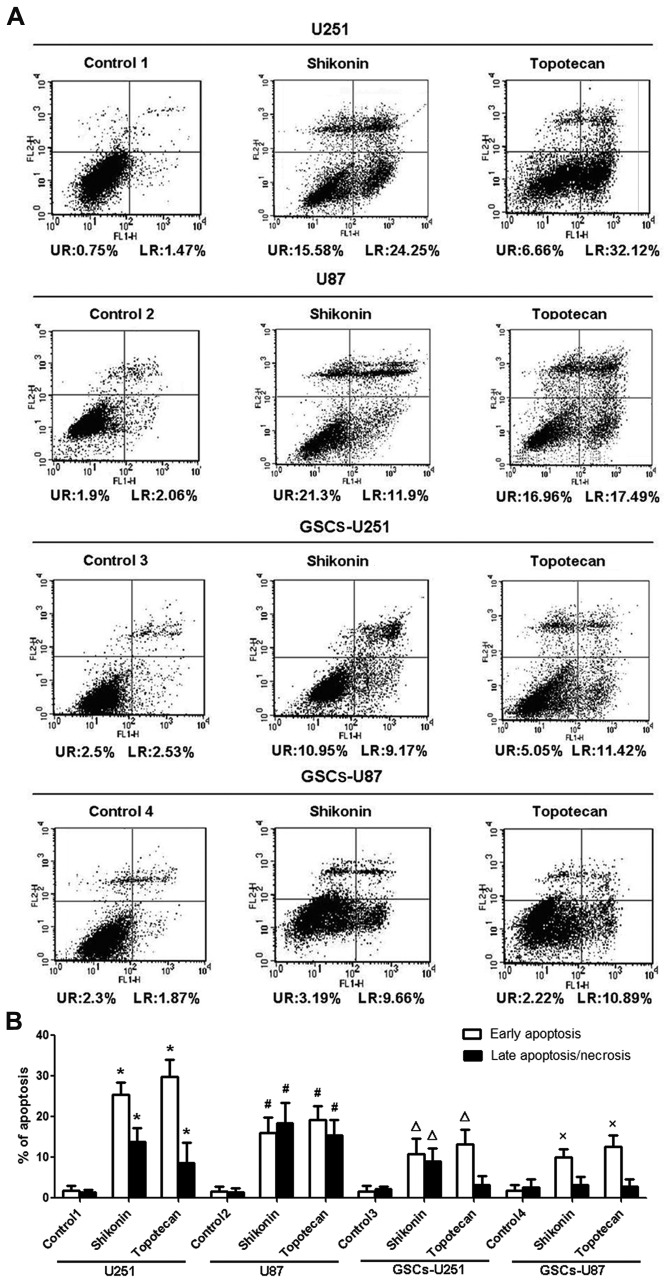
Shikonin or topotecan induced cell apoptosis. (A) Flow cytometric dot plots of Annexin V and PI staining. (B) Quantitative analysis of the early and late apoptosis rate. Data are given as mean±SD and are representative of four separate experiments. Control groups 1 to 4 were U251, U87, GSCs-U251 and GSCs-U87 cells treated with the same amount of culture medium. *P<0.01 versus control group 1; ^#^ *P*<0.01 versus control group 2; ^Δ^ *P*<0.01 versus control group 3; ^×^ *P*<0.01 versus control group 4.

Annexin V and PI staining for flow cytometry was used to detect apoptosis in U251, U87, GSCs-U251 and GSCs-U87 cells treated with shikonin or topotecan for 24 h. The results of Figure 4 showed that the percentage of apoptotic U251 cells in control group was 2.35±1.94%, and was significantly increased to 39.28±3.21% and 38.60±3.08% in shikonin and topotecan groups respectively. In U87 cells, the apoptotic percentage of control group was 2.26±1.18%, and was increased to 33.64±5.98% and 34.26±5.34% markedly in shikonin and topotecan group, respectively. In GSCs-U251 cells, the apoptotic percentage of control group was 4.15±1.56%, while those of shikonin and topotecan groups were 20.68±2.16% and 17.36±4.42%, respectively. In GSCs-U87 cells, the apoptotic percentage of control group 4 was 4.33±2.43%, and those of shikonin and topotecan groups were 13.5±3.2% and 14.14±3.01%, respectively. The apoptosis induced by shikonin in U251 and GSCs-U87 after 24 h was in the early stage; while the treatment of topotecan after 24 h induced the early apoptosis of U251, GSCs-U251 and GSCs-U87 as well. The above results showed that shikonin or topotecan significantly induced apoptosis in different glioma cells and GSCs compared to the control groups.

### Shikonin or Topotecan Decreases Bcl-2 Levels and Increases Cleaved Caspase-9 and -3 Levels in GSCs

**Figure 5:**
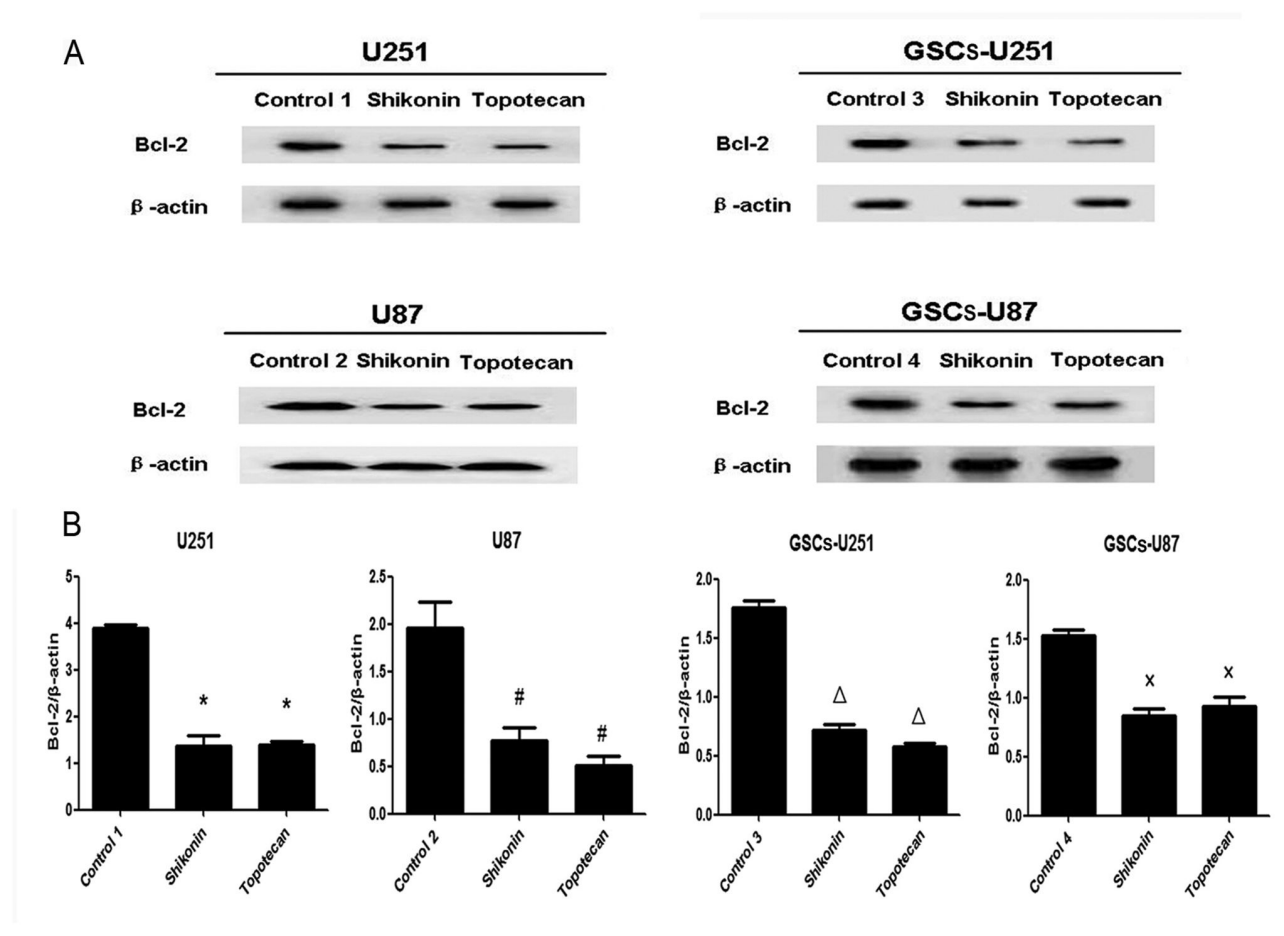
Shikonin or topotecan decreased Bcl-2 protein expression. The Bcl-2 expressing levels (A) and quantitative analysis (B) of U251, U87, GSCs-U251 and GSCs-U87 cells treated with shikonin or topotecan. β-actin was used as a loading control. Data are given as mean±SD and are representative of three separate experiments. Control groups 1 to 4 were U251, U87, GSCs-U251 and GSCs-U87 cells treated with the same amount of culture medium. *P<0.01 versus control group 1; ^#^ *P*<0.01 versus control group 2; ^Δ^ *P*<0.01 versus control group 3; ^×^ *P*<0.01 versus control group 4.

**Figure 6:**
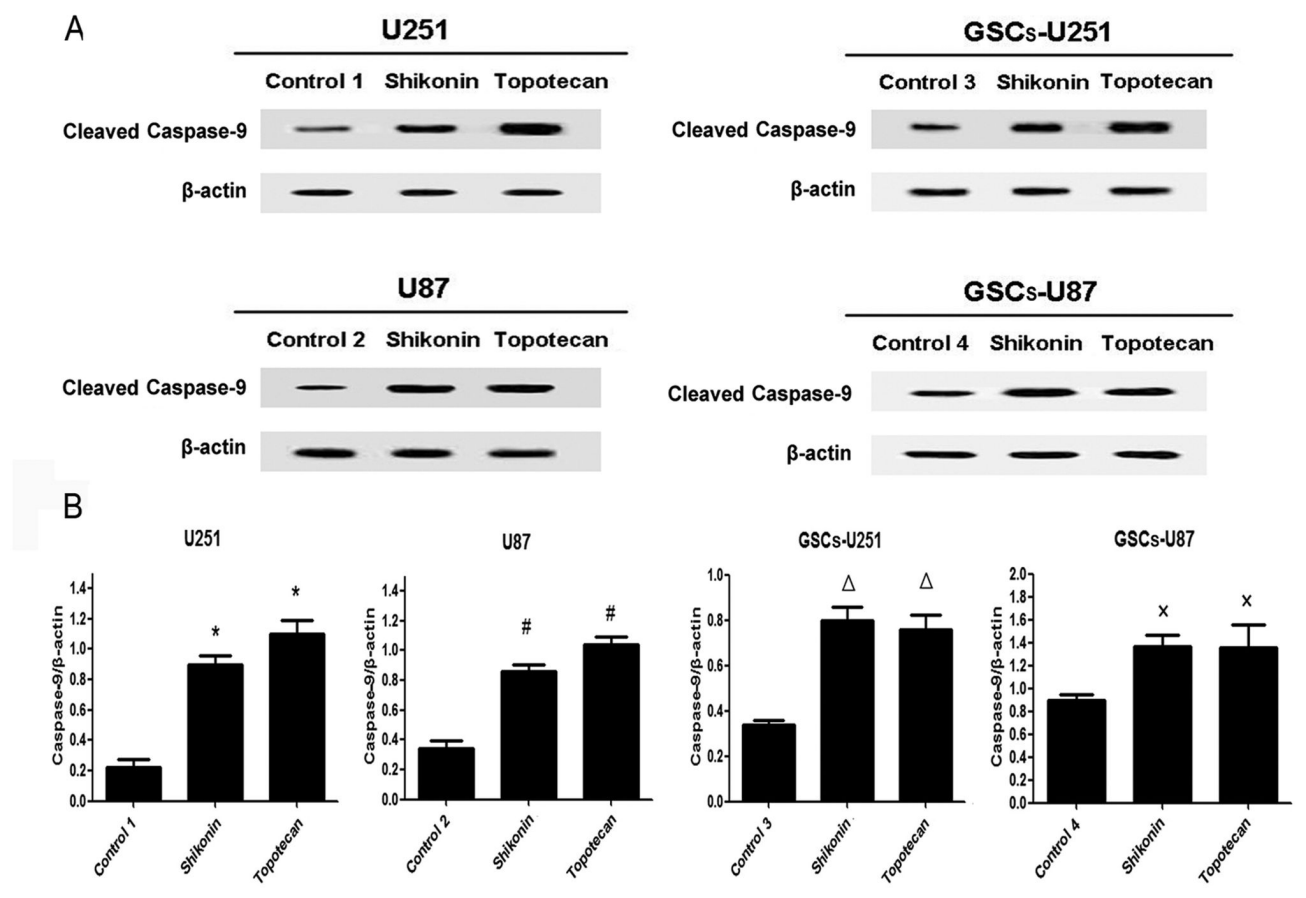
Shikonin or topotecan upregulated cleaved caspase-9 protein expression. The cleaved caspase-9 expressing levels (A) and quantitative analysis (B) of U251, U87, GSCs-U251 and GSCs-U87 cells treated with shikonin or topotecan. β-actin was used as a loading control. Data are given as mean±SD and are representative of three separate experiments. Control groups 1 to 4 were U251, U87, GSCs-U251 and GSCs-U87 cells treated with the same amount of culture medium. *P<0.01 versus control group 1; ^#^ *P*<0.01versus control group 2; ^Δ^ *P*<0.01versus control group 3; ^×^ *P*<0.01 versus control group 4.

**Figure 7:**
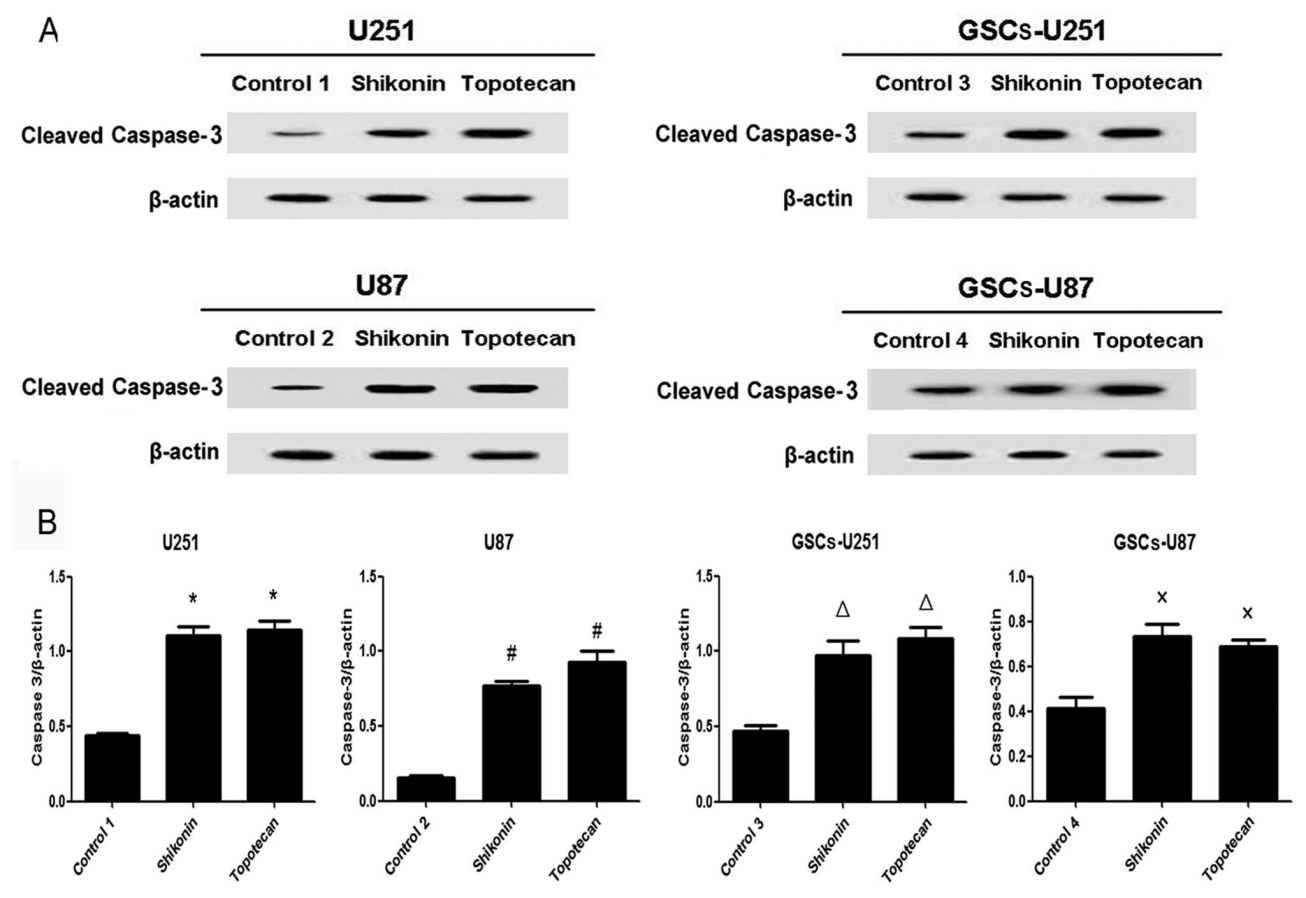
Shikonin or topotecan increased cleaved caspase-3 protein expression. The cleaved caspase-3 expressing levels (A) and quantitative analysis (B) of U251, U87, GSCs-U251 and GSCs-U87 cells treated with shikonin or topotecan. β-actin was used as a loading control. Data are given as mean±SD and are representative of three separate experiments. Control groups 1 to 4 were U251, U87, GSCs-U251 and GSCs-U87 cells treated with the same amount of culture medium. *P<0.01 versus control group 1; ^#^ *P*<0.01 versus control group 2; ^Δ^ *P*<0.01 versus control group 3; ^×^ *P*<0.01 versus control group 4.

U251, U87, GSCs-U251 and GSCs-U87 cells were treated with shikonin or topotecan for 24 h and the protein expressing levels of Bcl-2 (Figure 5), cleaved caspase-9 (Figure 6) and cleaved caspase-3 (Figure 7) were detected by western blot assay. In U251 and U87 cells, the expression of Bcl-2 was decreased significantly, meanwhile the activity of caspase-9 and caspase-3 were elevated significantly (*P*<0.01), compared to the control groups. Similar with the results of glioma cells, shikonin or topotecan significantly reduced the expression of Bcl-2 as well as promoted the activation of caspase-9 and caspase-3 in GSCs-U251 and GSCs-U87 cells respectively, compared to the control groups (*P*<0.01). Cisplatin, a caspase activator, was used to induce apoptosis as a positive control compared with non treatment control. The results indicated that after treatment of cisplatin (5 μg/ml) for 24 h, the protein levels of cleaved caspase-3 increased by 3-fold in GSCs, which was higher than those (approximately 2-fold) induced by shikonin and topotecan (Figure S1).

## Discussion

According to recent researches, GSCs may be the initiating cells in glioblastoma and have currently gained attention as the therapeutic target. In this experiment, we revealed that shikonin or topotecan inhibited the proliferation of the GSCs as well as the glioma cells in a dose- and time-dependent manner. In addition, shikonin and topotecan induced cell cycle arrest in G0/G1 and S phases and promoted apoptosis. The down-regulation of Bcl-2 and activation of caspase 9/3-dependent pathway were involved in the apoptosis process. We demonstrated for the first time that shikonin and topotecan might be the potential anticancer drugs targeting GSCs.

GSCs are defined as a small population of cells capable of extensive proliferation, self-renewal, multipotent differentiation and tumor initiation. Methodologically, it has been difficult to isolate and amplify GSCs from established tumor cell lines. Usually, the isolation and identification of GSCs involve two types of methods: one is based on the sorting of side population (SP) cells that can exclude the hoechst 33342 dye. However, Hoechst 33342 is cytotoxic; consequently, SP cells are protected by their membrane transporter properties, whereas unprotected non-SP cells suffer toxicity and are unable to grow. Thus the differing tumor-initiation abilities of SP and non-SP cells are most likely due to an artifact of Hoechst 33342 toxicity, rather than due to intrinsic stem-cell properties. Moreover, there are conflicting data showing that either the sorted SP or non-SP cells were similarly clonogenic in vitro and equally tumorigenic in vivo. In addition, some protein transporter’s function is lost in glioma endothelial cells, correlating with the blood brain barrier loss of integrity seen in glioma patients [19,20]. The other type includes the fluorescence activated cell sorting and the magnetic activated cell sorting, which are based on glioma cell surface markers include CD133, CD15, L1CAM, Integrin α6. Due in large part to conflicting results and irreproducibility of experiments, a lot of disagreement exists regarding the use of a specific marker or a combination of different markers to identify and isolate GSCs [21]. Recently, sphere culture has been increasingly used as a method for enriching stem cells which relies on their property of anchorage independent growth. We and other researchers have reported the application of sphere culture to isolate, enrich, maintain or expand potential GSC subpopulations from human glioma cell lines, such as U373, A172 and U87. The accumulating evidence showed that GSCs could be cultivated in vitro in EGF- and bFGF-enriched serum-free medium, because EGF and bFGF induce proliferation of multipotent, self-renweing, and expandable GSCs. The resulting cellular spheroids were positive for CD133 and other putative stem cell markers like nestin. When cultured in differentiation medium containing serum, the CD133^+^ cells differentiate into neural cell lineages [22-24]. In our experiment, cellular spheroids bearing cancer stem-like characteristics such as self-renewal and multipotency were successfully isolated from U251 and U87 glioma cells. Immunofluorescence staining showed that the cells within the sphere expressed CD133 and nestin, the markers of neural stem cell, which indicated that these cells had self-renewal ability. The percentage of CD133^+^ cells were 98.2% in GSCs-U251 and 98.6% in GSCs-U87, which were similar with those confirmed in glioma-initiating cells isolated from U87 and 4910 glioma xenograft cells (98.4% and 98.8%, respectively) using the same isolated method [25]. The neuron-specific cytoskeletal protein β-tubulin-III and astrocyte-specific cytoskeleton protein GFAP were found in the differentiated cells, which suggested that the cellular spheroids were multipotent for at least two neural cell types. These results are in accordance with the studies about cellular spheroids from the human glioma cell lines [26,27].

Shikonin or topotecan is commonly used in tumor therapy. Shikonin has been reported to have potent anti-tumor effects against various tumors, such as osteosarcoma, leukemia, breast cancer, carcinoma of salivary gland and so on [28-31]. Topotecan is a traditional antineoplastic drug. Some recent studies clarified that topotecan in combination with other chemotherapeutic agents played an important role in combating drug resistant of glioblastoma multiforme [32]. To investigate the anti-cancer effects of shikonin or topotecan on glioma cell lines and GSCs, various doses of the two drugs were administrated in U251, U87, GSCs-U251 and GSCs-U87 cells, respectively, in our research. The results demonstrated that both shikonin and topotecan were able to inhibit proliferation of human glioma cells and GSCs in a dose- and time-dependent manner. According to the IC50 values at 24 h, 2 μmol/L of shikonin and 3 μmol/L of topotecan were selected as the optimal administration concentration to investigate their effects on glioma cells and GSCs. Shikonin and topotecan displayed better cytotoxicity in glioma cells (average IC50: 1.93 μmol/L and 2.84 μmol/L, respectively) than GSCs with average IC50 values of 3.84 μmol/L and 5.71 μmol/L, respectively.

Previous studies showed that shikonin and topotecan inhibited tumor growth through inducing cell cycle arrest. Chin-Chung Yeh, et al demonstrated that shikonin (16 μmol/L) increased the percentage of the T24 human bladder cancer cells in G0/G1-phase, and decreased the percentage of the cells in S, G2/M-phases [18]. Yingkun, et al suggested that shikonin inhibited HepG2 cell growth in a dose-dependent manner and blocked the S phase progression [33]. In this experiment, we also proved that the treatment of shikonin (2 μmol/L) for 24 h induced glioma cells and GSCs cell cycle arrest in G0/G1 and S phases, therefore inhibited tumor cells growth. Several experiments have revealed that topotecan affected the cell cycle. Francesca, et al found that 4 μmol/L of topotecan induced a late-middle S-phase cell cycle arrest in H526 cells [34]. Similarly, our data showed topotecan (3 μmol/L) significantly increased the percentage of the glioma cells and GSCs in the G0/G1 and S phase, decreased those of the G2/M stage, which suggested that topotecan induced arrest in the G0/G1 and S stage. Meanwhile, the obvious sub-G1 population appeared to the left of the G0/G1 phase, which indicated that shikonin or topotecan could induce the apoptosis of glioma cells and GSCs. Because shikonin induced necrosis could be transformed into apoptosis [35,36], the percentage of apoptotic cells by Annexin V/PI analysis was higher than that by cell cycle assay. Ching-Hsein Chen, et al suggested that shikonin (7.5 μmol/L) did not induce marked apoptosis in human astrocytes (sub-G1<6%), a normal cell line, indicating that glioma cells, but not normal cells, were sensitive to shikonin [37]. After treatment with 3 μmol/L of topotecan for 24 h, the percentage of apoptotic cells by Annexin V/PI analysis was higher than that by cell cycle assay as well. The Topo I suppressor shikonin and the Topo I poison topotecan are well known as chemotherapeutic agents, however, few works have investigated the anti-cancer effects of these two classes of Topo I inhibitor on GSCs. Our data demonstrated that shikonin or topotecan induced GSCs cell cycle arrest in G0/G1 and S phases as well as promoted GSCs apoptosis.

The down-regulation of anti-apoptotic protein Bcl-2 and activation of caspase 9/3-dependent pathway were associated with tumor cell growth inhibition, especially in glioma cells [38,39]. In this study, after the treatment of shikonin or topotecan on the GSCs as well as glioma cells for 24 h, the expression of Bcl-2 protein significantly decreased, meanwhile the cleaved caspase-9 and cleaved caspase-3 proteins increased to a higher level, which indicated that inhibition of Bcl-2 protein with activation of caspase 9/3-dependent pathway was involved in shikonin or topotecan induced apoptosis process. These results are consistent with previous report that shikonin derivative or topotecan may induce apoptosis of cancer cell lines through caspase pathway, similar cleavage of caspase-9 and caspase-3 was observed in this process [40,41]. Though GSCs were resistant to some anti-cancer agents, this study proved that shikonin or topotecan had a significant inhibitory effect on the proliferation of GSCs and had the potential for clinical administration.

In conclusion, our current research demonstrated for the first time that shikonin or topotecan significantly inhibited the cell viability, induced cell cycle arrest in G0/G1 and S stage and promoted apoptosis of GSCs as well as glioma cells. The down-regulation of Bcl-2 expression with activation of caspase 9/3-dependent pathway was involved in these processes. Topo I inhibitors may be the potential drugs targeting GSCs that will open up new opportunities to develop therapeutic strategies.

## Supporting Information

Figure S1 **Cisplatin increased cleaved caspase-3 protein expression.** The cleaved caspase-3 expressing levels of U251, U87, GSCs-U251 and GSCs-U87 cells induced by cisplatin (5 μg/ml) after 24 h was analyzed by western blot. Data are given as mean±SD and are representative of three separate experiments. *P<0.01 versus the corresponding control group.
(TIF)
