# Supplementary figures and images for: Topoisomerase I Inhibitors, Shikonin and Topotecan, Inhibit Growth and Induce Apoptosis of Glioma Cells and Glioma Stem Cells
Source: PLoS One. 2013 Nov 26;8(11):e81815. doi: 10.1371/journal.pone.0081815 (PMC3841142; doi:10.1371/journal.pone.0081815)

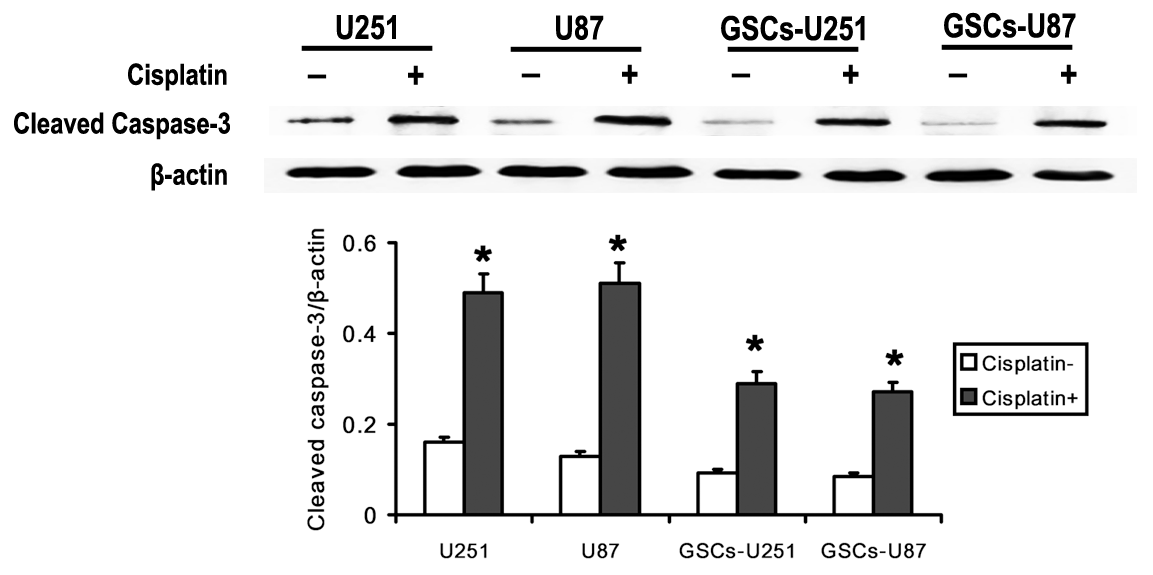

Supplement: Figure S1 — Cisplatin increased cleaved caspase-3 protein expression. The cleaved caspase-3 expressing levels of U251, U87, GSCs-U251 and GSCs-U87 cells induced by cisplatin (5 μg/ml) after 24 h was analyzed by western blot. Data are given as mean±SD and are representative of three separate experiments. *P<0.01 versus the corresponding control group. (TIF) [file pone.0081815.s001.tif]
